# An Interactive Human-in-the-Loop Framework for Skeleton-Based Posture Recognition in Model Education

**DOI:** 10.3390/biomimetics10070431

**Published:** 2025-07-01

**Authors:** Jing Shen, Ling Chen, Xiaotong He, Chuanlin Zuo, Xiangjun Li, Lin Dong

**Affiliations:** 1 College of Engineering and Design, Hunan Normal University, Changsha 410081, China; 2 Institute of General and Applied Linguistics and Phonetics (ILPGA), Sorbonne Nouvelle University—Paris 3, 75012 Paris, France; 3 IBISC Laboratory, University of Paris Saclay, Évry, 91000 Paris, France; 20235232@etud.univ-evry.fr (C.Z.);; 4 Institute of Sports Artificial Intelligence, Capital University of Physical Education and Sports, Beijing 100191, China

**Keywords:** skeleton-based posture recognition, deep learning, human pose estimation, human-in-loop-control, intelligent teaching system

## Abstract

This paper presents a human-in-the-loop interactive framework for skeleton-based posture recognition, designed to support model training and artistic education. A total of 4870 labeled images are used for training and validation, and 500 images are reserved for testing across five core posture categories: standing, sitting, jumping, crouching, and lying. From each image, comprehensive skeletal features are extracted, including joint coordinates, angles, limb lengths, and symmetry metrics. Multiple classification algorithms—traditional (KNN, SVM, Random Forest) and deep learning-based (LSTM, Transformer)—are compared to identify effective combinations of features and models. Experimental results show that deep learning models achieve superior accuracy on complex postures, while traditional models remain competitive with low-dimensional features. Beyond classification, the system integrates posture recognition with a visual recommendation module. Recognized poses are used to retrieve matched examples from a reference library, allowing instructors to browse and select posture suggestions for learners. This semi-automated feedback loop enhances teaching interactivity and efficiency. Among all evaluated methods, the Transformer model achieved the best accuracy of 92.7% on the dataset, demonstrating the effectiveness of our closed-loop framework in supporting pose classification and model training. The proposed framework contributes both algorithmic insights and a novel application design for posture-driven educational support systems.

## 1. Introduction

The realm of arts education, a critical facet of contemporary pedagogical landscapes, increasingly recognizes the pivotal role of human posture recognition and classification. This technological advancement not only reshapes the teaching dynamics across various art forms but also holds particular significance in model education. In this context, where presentation, poise, and physical expression are paramount, the precise understanding and replication of human postures transcend traditional teaching methodologies. By integrating advanced posture recognition systems, educators can enhance instructional strategies, enabling a more nuanced and accurate representation of human form and expression. This convergence of technology and pedagogy marks a transformative step in the evolution of arts education, fostering a more profound learning experience [1,2,3].

Historically, human posture recognition technologies have undergone significant evolution, marked by distinct phases of technological advancement and inherent limitations. Early systems primarily relied on traditional image processing techniques, which focused on extracting key points and contours from two-dimensional images [4,5,6]. While these pioneering methods laid the foundation for the field, they were constrained by limited computational power and the inability to handle complex environmental variables [7]. The introduction of computer vision methodologies significantly improved the accuracy and robustness of posture recognition systems by incorporating feature point detection, model matching, and skeletal representation. These methods represented a notable shift, enabling more reliable recognition in controlled environments. However, the integration of deep learning technologies, such as Convolutional Neural Networks (CNNs) and Recurrent Neural Networks (RNNs), marked the most transformative phase. Deep learning allowed systems to automatically extract complex features from large-scale datasets [8,9,10], thereby enhancing their adaptability to real-world, dynamic scenarios.

The rapid development of deep learning has catalyzed the transition from static motion tracking to real-time, multi-dimensional posture recognition. CNNs excel in processing spatial information, while RNNs and Long Short-Term Memory (LSTM) networks are particularly effective for sequential data analysis [11]. More recently, Transformer-based architectures have demonstrated superior performance in learning long-range dependencies, offering new possibilities for understanding complex human motion sequences [12]. These advancements have paved the way for more robust posture recognition systems that can discern subtle gestures and body movements with high accuracy, even in unstructured environments. Meanwhile, human-in-the-loop frameworks have emerged as a promising way to introduce expert knowledge and iterative supervision, especially in education and training applications.

In the context of model education, posture recognition and classification technologies are indispensable for developing technical skills and enhancing artistic expression. Accurate posture classification provides objective and data-driven insights into body positioning, movement efficiency, and balance. Traditional teaching approaches rely heavily on subjective observation and delayed feedback, which can hinder the learning process and reduce instructional precision. By contrast, advanced posture recognition systems enable real-time feedback, allowing students to refine their postures and movements iteratively and efficiently [13,14]. This interactive [15] learning environment fosters personalized instruction, enabling students to explore different postures and receive immediate corrections, thus improving both their technical proficiency and creative expression.

Furthermore, the application of posture recognition extends beyond the educational setting to a variety of domains, including human-computer interaction, robotics collaboration, motion-sensing games, and sports performance analysis [16,17,18,19]. These diverse applications underscore the versatility and robustness of posture recognition technology, proving its potential to revolutionize arts education. In model education, where body language and physical expression are integral to artistic performance, posture recognition systems provide an unprecedented level of precision and adaptability. By offering real-time analysis and feedback, these systems allow educators and students to dissect complex body movements, ensuring that even the most intricate postural nuances are accurately captured and analyzed.

Despite the promising developments in posture recognition, several challenges remain in identifying the most effective combination of features and classification algorithms for different postural contexts. Commonly used features include joint coordinates, angular measurements, limb length ratios, and body symmetry [20], each offering unique advantages. Selecting the optimal feature combination is critical for achieving high classification accuracy while maintaining computational efficiency. Equally important is the choice of classification algorithm. Traditional machine learning models, such as K-Nearest Neighbors (KNN), Support Vector Machine (SVM), and Random Forest, have shown efficacy in static posture classification [21], whereas deep learning architectures like LSTM and Transformer networks excel in recognizing dynamic posture sequences [22]. Balancing model complexity, accuracy, and computational requirements is a key consideration in building practical posture recognition systems for real-world applications.

Building on this foundation, this study proposes a comprehensive framework for multi-feature skeleton-based posture recognition and classification in model education. The primary objective is to evaluate the performance of different feature combinations and classification algorithms, identifying the most effective strategies for various postural categories. Extensive experiments are conducted on a custom dataset of model postures, covering common poses such as standing, sitting, jumping, crouching, and lying. The results provide valuable insights into how specific feature combinations influence classification accuracy and computational complexity. Finally, we introduce a real-time posture recommendation system that leverages classification results to offer personalized learning suggestions for model trainees. This system aims to bridge the gap between traditional teaching methods and intelligent learning technologies, fostering a more interactive and effective learning environment.

## 2. Related Works

Human posture recognition is a compelling and complex subject within the domain of computer vision, owing to its extensive applicability across various fields. Over the years, continuous research advancements have led to the emergence of numerous technologies for human pose recognition, each representing significant milestones in the evolution of this domain. This section provides an overview of these developments, focusing on sensor-based systems, artificial neural networks, and computer vision techniques, while emphasizing their diverse applications and unique contributions, especially in the context of model education.

Innovative sensor technologies have significantly influenced the landscape of human posture recognition, offering enhanced accuracy and adaptability across a broad range of contexts. Notably, the integration of Microsoft Kinect into posture recognition systems by Zhang et al. [23] represented a major breakthrough by enabling automatic identification of user-defined postures with high accuracy, enhanced by machine learning techniques that replaced the need for empirical thresholds [24,25]. This approach was further advanced by invasive systems employing MPU6050-based sensors, as introduced by Kale et al. [16], where accelerometer data from sensors placed on the chest and thigh was transmitted wirelessly to a Raspberry Pi for real-time processing, achieving a remarkable accuracy rate of 97.589%. Such sensor-based methodologies have been particularly effective in healthcare applications. Additionally, Liu et al. [26] proposed a motion capture system based on MEMS sensors and Zigbee networks, which demonstrated a 10% increase in system efficiency and a 15% improvement in accuracy, further validating the practical advantages of sensor-based solutions for real-time human posture monitoring.

Recent advancements in artificial neural networks (ANNs) have revolutionized human posture recognition by improving precision and efficiency, particularly in scenarios involving large datasets or complex motion patterns. A notable example is the work by Núñez et al. [27], which combined Convolutional Neural Networks (CNNs) and Long Short-Term Memory (LSTM) networks for human activity and hand gesture recognition using 3D skeleton data. Their two-stage training strategy and validated data augmentation techniques significantly improved performance, especially for small datasets. Similarly, Ding et al. [28] developed a 219-dimensional vector representation incorporating angle and distance features for human posture recognition, utilizing rule learning, Bagging, and random subspace methods to optimize sub-classifier performance. Another innovative approach [29] leveraged fuzzy logic and machine learning to recognize fine-grained lying postures in healthcare robotics, contributing to patient safety through precise posture identification. A cutting-edge hybrid classifier introduced in 2020 combined an adaptive signal segmentation algorithm with a multi-layer perceptron, achieving real-time posture recognition with superior accuracy compared to single classifiers [30].

In parallel, computer vision and deep learning techniques have played an increasingly pivotal role in advancing human posture recognition. Bantupalli et al. [31] developed a vision-based application for sign language translation by integrating Inception-based Convolutional Neural Networks with a Recurrent Neural Network (RNN) to extract spatial and temporal features from video sequences, significantly improving communication between signers and non-signers. Building on these advancements, Kulikajevas et al. [32] introduced a Deep Recurrent Hierarchical Network (DRHN) model based on MobileNetV2 for human posture detection, addressing the challenge of occlusion and achieving an accuracy rate of 91.47% on RGB-Depth frame sequences. More recently, Ogundokun et al. [33] proposed a hybrid model combining InceptionV3 Deep Convolutional Neural Networks and Support Vector Machines (SVM), achieving an accuracy rate of 95.42% by employing LASSO-based feature selection and various regularization techniques to prevent overfitting.

In the realm of performing arts, computer vision systems have been extensively applied for gesture recognition and choreography analysis. Bakalos et al. [34] developed a system for recognizing finger musical gestures in 3D space, enabling performance without tangible instruments through image analysis and machine learning techniques such as Hidden Markov Models and Gaussian Mixture Models. Similarly, 3D motion capture technologies, including Microsoft Kinect and Vicon systems, have been utilized to preserve Intangible Cultural Heritage (ICH) by digitizing and analyzing traditional dance movements. Zhou et al. [35] illustrated the application of embedded artificial intelligence for analyzing dance postures in traditional Chinese dance, highlighting the potential of AI in cultural preservation and performance enhancement. These technologies underscore the growing importance of visual analysis in the performing arts, offering tools to both preserve and modernize traditional practices.

The application of visual technologies in model education holds significant potential for enhancing training outcomes and professional development. These technologies encompass a wide range of tools, including video analysis, motion capture, and real-time feedback systems, each contributing to improved performance assessment and skill refinement. Video analysis [36] enables comprehensive reviews of modeling performances, helping both models and coaches identify areas for improvement. Posture recognition and detection technologies ensure that poses meet professional standards, while real-time feedback systems offer instant guidance to rectify errors and optimize performance. Virtual reality [37] provides an immersive training environment where models can practice and perfect their skills with reduced risk. Motion capture systems equipped with real-time feedback enhance the precision of training, allowing for immediate corrections during live practice sessions. These advancements not only improve modeling skills but also reinforce professionalism, adaptability, and competitiveness in the fashion industry. As such, the integration of visual technologies into model education carries practical significance and substantial value for the long-term success of models.

In summary, human posture recognition has evolved from sensor-based systems to deep learning-driven approaches, each contributing unique advantages across various domains. The increasing convergence of these technologies with performing arts and model education highlights their transformative potential. This paper builds upon these advancements by proposing a multi-feature skeleton-based posture recognition framework. Unlike traditional applications primarily focused on classification or surveillance, our approach emphasizes its novel use in model education, where real-time posture recognition supports model training and teaching. By enabling efficient pose matching and human-in-the-loop interaction [38], the system enhances instructional quality and learning efficiency. The framework focuses on optimal feature extraction, classification accuracy, and real-world adaptability, ultimately aiming to improve personalized pose recommendation and artistic expression in model education settings.

## 3. Methodology

### 3.1. Interactive Posture Recommendation System for Model Training and Teaching

To address the practical need for efficient and personalized posture guidance in model training and artistic education, we design an interactive posture recommendation system that integrates skeleton-based pose classification with a human-in-the-loop visual suggestion workflow. This system aims not only to recognize the pose of the model in real time but also to support instructors in selecting optimal reference images [39] for teaching and imitation.

**Figure 1:**
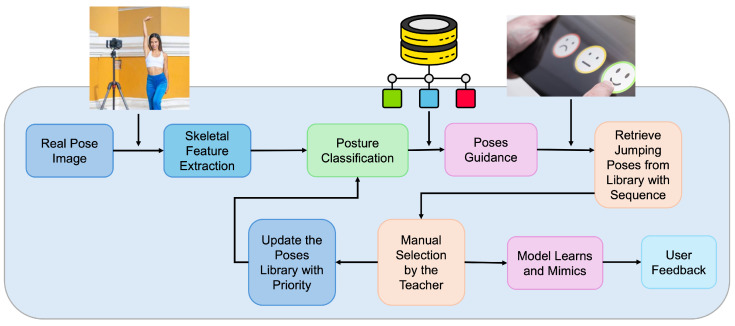
Overview of the proposed human-in-the-loop interactive posture recommendation system for model training.

As illustrated in Figure 1, the process begins with the capture of a real-time image of the model using a camera. The input image is processed using a skeleton detection framework (e.g., MediaPipe Pose), and keypoint features such as joint coordinates, angles, limb symmetry, and body proportions are extracted. These features are then input into a trained classifier (e.g., Transformer or LSTM-based model) to determine the posture category among five fundamental actions: standing, sitting, jumping, lying, and crouching. Once the pose is classified, the system retrieves a set of relevant reference poses from a pre-tagged posture library. These reference images are organized by category and annotated with quality indicators such as symmetry score or expressiveness rating. The retrieved images are displayed through a visual interface, where the teacher can swipe through and manually select the most suitable example for demonstration.

This human-in-the-loop selection mechanism ensures that the system provides computational efficiency while retaining artistic control and subjective judgment. The chosen reference is then presented to the model learner, who attempts to mimic the pose. In this way, the system offers both real-time classification and curated inspiration for modeling posture training. Moreover, feedback from the teacher’s selections and model performance can be used to update the system’s pose library. For example, frequently selected poses may be prioritized in future suggestions, and user-corrected outputs can help refine classifier decision boundaries. This closed-loop interaction [40] between model, machine, and teacher creates a dynamic and adaptive teaching assistant that enhances traditional training with computational guidance. Compared to conventional skeleton-based classification systems, our framework introduces a new layer of applied functionality: not merely labeling a pose, but supporting an interactive, visual posture recommendation process that improves the quality, efficiency, and personalization of model education.

### 3.2. Data Collection and Dataset Description

The dataset used in this study consists of 4870 labeled images of human postures for training and 500 for testing, covering five distinct categories: standing, sitting, jumping, lying, and crouching. These pose categories represent foundational positions frequently utilized in model training and performing arts, reflecting both static and dynamic scenarios.

To ensure the quality and consistency of the dataset, images were collected under controlled lighting and minimal occlusion environments. To enhance variability and improve model generalization, data augmentation techniques were employed, including controlled variations in rotation, scale, and light occlusion. These augmentations simulate real-world conditions and strengthen the robustness of the classification models.

**Figure 2:**
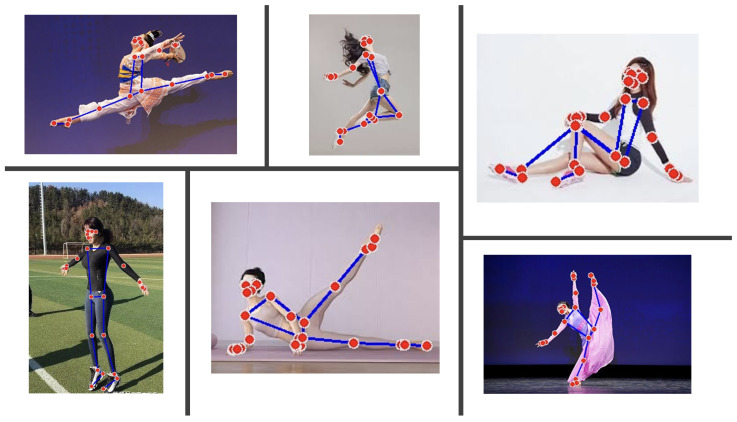
Visualization of Various Human Postures Captured in the Dataset with Skeletal Keypoint Representation.

Figure 2 presents several posture examples annotated with skeletal keypoints extracted using the MediaPipe Pose framework. These visualizations demonstrate the diversity of pose configurations included in the dataset and the complexity of spatial relationships captured by skeletal features. Each keypoint represents a key anatomical landmark used in calculating joint angles, limb distances, and body symmetry metrics.

The skeletal data for each image was extracted using the MediaPipe Pose framework [41], which identifies 33 anatomical keypoints per subject. Each keypoint $\left(x_{i},y_{i}\right)$ is normalized within the image coordinate space. These keypoints are structured into semantically meaningful segments—upper limbs, lower limbs, torso, etc.—forming the structural basis for feature extraction. A connectivity illustration of these keypoints is provided, demonstrating how spatial and geometric relationships among joints are used for feature engineering. These extracted features enable rich and informative representations of posture useful for downstream classification tasks.

### 3.3. Feature Extraction

The success of posture recognition depends largely on the quality and variety of features extracted from skeletal data. To fully capture human posture characteristics, we compute several types of features, including joint coordinates, joint angles, limb lengths, and symmetry scores. These features complement each other by capturing spatial position, geometric relationships, and pose balance. The normalized joint coordinates $\left(x_{i},y_{i}\right)$ for all 33 keypoints form the base feature set:

(1) $$C=\{\left(x_{i},y_{i}\right)\;|\;i=1,2,\ldots ,33\}.$$

These coordinates describe the skeleton’s structure and support the derivation of higher-order features like angles and distances.

Joint angles are important for understanding orientation of limbs and posture intent. Given three keypoints $A(x_{1},y_{1})$, $B(x_{2},y_{2})$, and $C(x_{3},y_{3})$, the angle θ at joint B is computed via the cosine law:

(2) $$\theta =\cos^{-1}\left(\frac{\mathrm{AB}\cdot \mathrm{BC}}{∥\mathrm{AB}∥∥\mathrm{BC}∥}\right),$$

where $\mathrm{AB}=(x_{2}-x_{1},y_{2}-y_{1})$, $\mathrm{BC}=(x_{3}-x_{2},y_{3}-y_{2})$. This angle is calculated at key joints like elbows, shoulders, hips, and knees.

To describe body proportions, we compute the limb length between two points $\left(x_{i},y_{i}\right)$ and $\left(x_{j},y_{j}\right)$ using:

(3) $$d_{ij}=\sqrt{{\left(x_{j}-x_{i}\right)}^{2}+{\left(y_{j}-y_{i}\right)}^{2}}.$$

For example:

$$\mathrm{Arm}\;\mathrm{Length}=d_{\text{shoulder-elbow}}+d_{\text{elbow-wrist}},$$

$$\mathrm{Leg}\;\mathrm{Length}=d_{\text{hip-knee}}+d_{\text{knee-ankle}}.$$

Symmetry plays an important role in posture quality and stability. To measure symmetry between left/right limbs, we use:

(4) $$S=\frac{|d_{\mathrm{left}}-d_{\mathrm{right}}|}{\max(d_{\mathrm{left}},d_{\mathrm{right}})}.$$

Smaller values of *S* mean better symmetry, while larger values indicate imbalance or dynamic motion.

To avoid redundant or irrelevant features and reduce dimensionality, we apply Principal Component Analysis (PCA) before classification. Given a dataset of *N* samples and *D* features, we first center the data:

(5) $$X_{c}=X-1\mu^{⊤},$$

where μ is the feature-wise mean and 1 is a column vector of ones.

Then, the covariance matrix is computed as:

(6) $$\Sigma =\frac{1}{N}X_{c}^{⊤}X_{c}.$$

Solving the eigenvalue decomposition:

(7) $$\Sigma v_{i}=\lambda_{i}v_{i},\;i=1,2,\ldots ,D,$$

we keep the top *k* eigenvectors such that:

(8) $$\sum_{i=1}^{k}\frac{\lambda_{i}}{\sum_{j=1}^{D}\lambda_{j}}\geq \eta ,$$

where η is the variance threshold (e.g., 95%).

The projection matrix $W\in R^{D\times k}$ is built from the top eigenvectors, and the reduced features are:

(9) $$Z=X_{c}W,$$

where Z is the final reduced feature matrix used for classification.

This process removes noise and reduces the input space to the most informative directions. In our case, it led to better classifier performance, lower training cost, and helped avoid overfitting, especially when using deep models with relatively limited posture data. The retained components still preserved the structural diversity needed for accurate recognition across different pose categories in model training scenarios.

### 3.4. Algorithmic Framework

**Figure 3:**
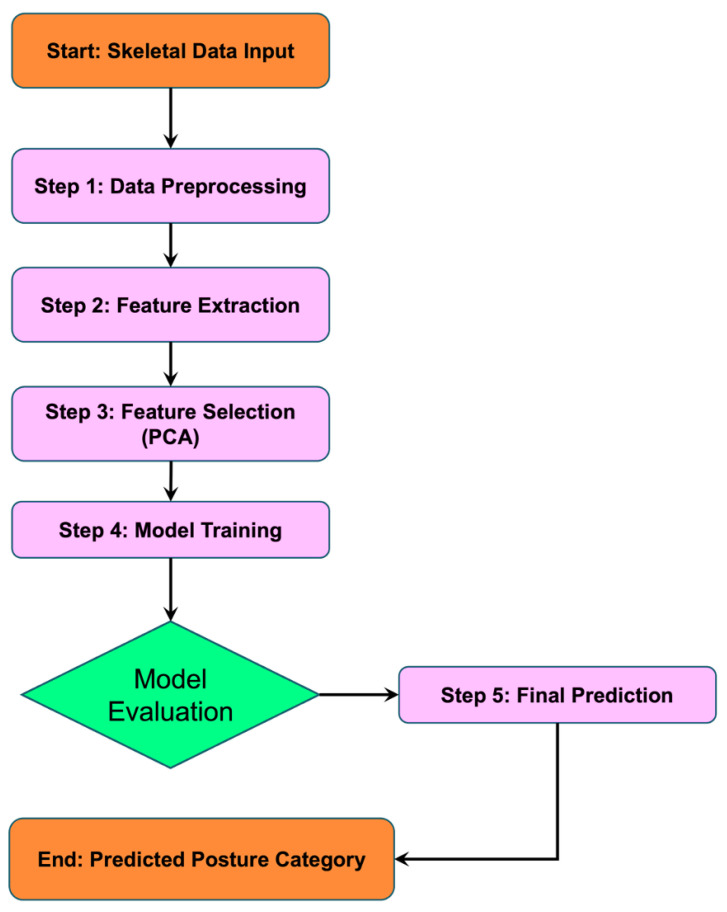
Proposed Framework Flowchart for Skeleton-based Posture Classification.

This section presents the algorithmic framework for skeleton-based human posture recognition and classification. The complete workflow is described in Algorithm 1, and the main stages are also visualized in Figure 3.
**Algorithm 1:** Proposed Multi-feature Skeleton-based Posture Classification Framework
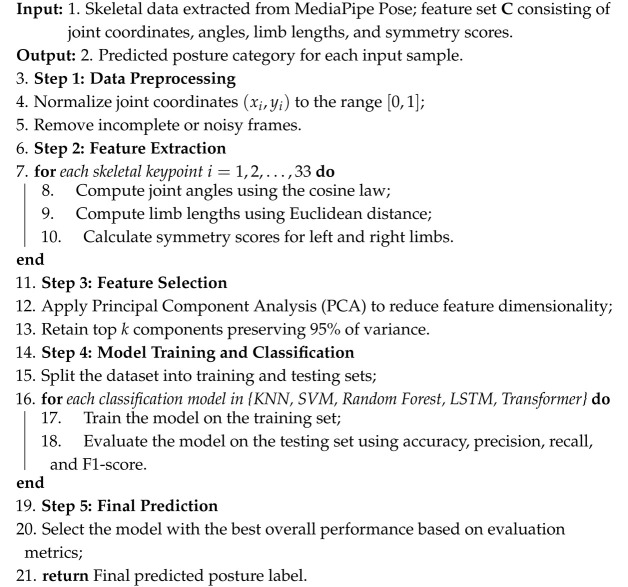


The proposed algorithm integrates both handcrafted features and learned representations, combining the benefits of classic models and neural networks. Using feature types that include geometric, spatial, and symmetric information, the system gains a broad view of posture characteristics.

Joint angles give information on body structure and orientation, while limb lengths and symmetry highlight proportion and balance, which are especially useful in modeling and performance settings. The PCA step reduces dimensionality without discarding meaningful variance, helping models run faster and generalize better.

The complete process begins with preprocessing and continues through feature computation, feature reduction, training, and prediction. Figure 3 outlines this pipeline visually, including key decisions like selecting the best model. In training, we test different classifiers, ranging from baseline models like KNN and SVM to more advanced ones such as LSTM and Transformer. Each has its own strengths—classical models handle static features well, while deep models like Transformer capture complex structures better.

This multi-model evaluation lets us better understand which types of classifiers perform well on this kind of data, especially under real-world variability. Performance metrics such as precision, recall, and F1-score are considered alongside accuracy to guide model selection. The selected model is then used for final inference.

This method is designed to support real-time posture applications such as interactive model teaching, where recognizing and guiding poses efficiently matters. With clear stages and explainable decisions, the system can also be expanded for use in choreography, fitness feedback, or motion coaching environments.

### 3.5. Model Configuration and Hyperparameter Optimization

The experimental setup involved configuring and tuning multiple classification models, combining traditional machine learning algorithms and deep learning architectures. Each model was optimized using cross-validation to achieve robust and reliable performance. This section details the model settings and the hyperparameter spaces used during optimization.

For K-Nearest Neighbors (KNN), the main hyperparameter is the number of neighbors (*k*), which determines how many surrounding data points influence the prediction. Based on the full dataset of 4870 samples, we varied *k* from 3 to 50 to better adapt to denser feature distributions. We also experimented with both uniform and distance-based weighting schemes. To further explore model sensitivity, we added Manhattan distance alongside the commonly used Euclidean metric. Results are summarized in Table 1.

For Support Vector Machines (SVM), both linear and radial basis function (RBF) kernels were tested. The regularization parameter (*C*) and kernel coefficient (γ) were varied to explore the trade-off between model complexity and fit. The values used in grid search are presented in Table 2.

The Random Forest classifier was configured with varying numbers of trees, tree depths, and minimum samples required for node splitting. This allowed control over overfitting and training stability. The settings are shown in Table 3.

In the deep learning setup, the LSTM model had two stacked layers with 128 hidden units each, followed by a dense layer with softmax activation. We tested batch sizes of 16, 32, and 64, along with learning rates from 0.001 to 0.0001 using the Adam optimizer. A dropout rate of 0.3 was applied, and early stopping with patience of 10 epochs was enabled to prevent overfitting.

For Transformer-based models, we used six attention layers and eight attention heads per layer. Cosine annealing and warm-up scheduling were applied to the learning rate. Similar to LSTM, we tested various batch sizes and dropout rates to tune performance. Table 4 lists all deep learning hyperparameter ranges.

Each model was evaluated on the test set using accuracy, precision, recall, and F1-score. Cross-validation ensured reliable estimation of generalization performance. For deep learning models, we also monitored GPU memory usage and training time to assess computational efficiency.

### 3.6. Experimental Setup and Evaluation Metrics

The experimental setup was designed to systematically evaluate the performance of both traditional machine learning and deep learning models on the proposed skeleton-based posture classification task. The dataset used in this study consists of a total of 5370 skeletal samples, including 4870 images used for training and 500 images reserved for testing. All samples were categorized into five posture classes: standing, sitting, crouching, jumping, and lying. The skeletal keypoints for each image were extracted using the MediaPipe Pose framework, resulting in 33 keypoints per sample. Each keypoint was represented by normalized coordinates $\left(x_{i},y_{i}\right)$ in the range [0,1], allowing for consistent comparison across subjects and image scales.

Several feature types were computed from the normalized keypoints to enrich the representation of human posture, including joint angles, limb lengths, and symmetry scores. Joint angles were calculated based on the cosine law applied to three consecutive keypoints, capturing relative body orientation. For three points $A\left(x_{1},y_{1}\right),B\left(x_{2},y_{2}\right),C\left(x_{3},y_{3}\right)$, the angle at point *B* is:

(10) $$\theta =\cos^{-1}\left(\frac{\mathrm{AB}\cdot \mathrm{BC}}{∥\mathrm{AB}∥∥\mathrm{BC}∥}\right),$$

with vectors AB and BC formed from the respective coordinates. Limb lengths were computed as Euclidean distances:

(11) $$d_{ij}=\sqrt{{\left(x_{j}-x_{i}\right)}^{2}+{\left(y_{j}-y_{i}\right)}^{2}},$$

and symmetry between corresponding limbs was assessed via the following ratio:

(12) $$S=\frac{|d_{\mathrm{left}}-d_{\mathrm{right}}|}{\max(d_{\mathrm{left}},d_{\mathrm{right}})},$$

providing an indicator of body alignment and balance.

Given the high dimensionality of the full feature set, Principal Component Analysis (PCA) was applied to reduce the dimensionality while preserving the most informative components. Let $X\in R^{n\times d}$ be the original feature matrix. The covariance matrix Σ was computed as:

(13) $$\Sigma =\frac{1}{n}X^{⊤}X,$$

and its eigenvalue decomposition provided the principal components. The top *k* eigenvectors corresponding to the largest eigenvalues were selected to ensure that 95% of the total variance was retained, enabling a more compact and robust representation.

All classification models were trained on the 4870-sample training set and evaluated on the 500-sample test set. To reduce performance variability and ensure reliable generalization, five-fold cross-validation was used during training. Each fold trained on 80% of the data and validated on the remaining 20%.

K-Nearest Neighbors (KNN), Support Vector Machine (SVM), and Random Forest were implemented using scikit-learn. For KNN, *k* was tested across a range from 3 to 50, with both uniform and distance-based weighting schemes. SVM models were tuned using grid search across different values of *C* (0.1 to 100) and γ (0.001 to 1), and both linear and RBF kernels were evaluated. Random Forest hyperparameters included number of trees (100, 200, 500), maximum depth (10, 20, 50), and minimum samples split (2, 5, 10).

In addition, deep learning models were implemented using TensorFlow v2.10 and PyTorch v1.12.1. LSTM networks were built with two stacked LSTM layers (128 units each) followed by softmax classification. The Adam optimizer was used, and learning rates between 0.001 and 0.0001 were explored. Batch sizes of 16, 32, and 64 were tested. Early stopping with a patience of 10 epochs was applied to avoid overfitting.

Transformer-based models were also examined, with a configuration of 6 attention layers and 8 heads per layer. A warm-up learning rate scheduler and cosine annealing were used. Dropout was applied with a rate of 0.2 to reduce overfitting, and batch sizes of 32 and 64 were tested.

Model performance was evaluated using accuracy, precision, recall, and F1-score. Accuracy is defined as:

(14) $$\mathrm{Accuracy}=\frac{\mathrm{TP}+\mathrm{TN}}{\mathrm{TP}+\mathrm{TN}+\mathrm{FP}+\mathrm{FN}},$$

precision and recall as:

(15) $$\mathrm{Precision}=\frac{\mathrm{TP}}{\mathrm{TP}+\mathrm{FP}},\;\mathrm{Recall}=\frac{\mathrm{TP}}{\mathrm{TP}+\mathrm{FN}},$$

and F1-score as:

(16) $$F1-\mathrm{Score}=2\cdot \frac{\mathrm{Precision}\cdot \mathrm{Recall}}{\mathrm{Precision}+\mathrm{Recall}}.$$

Training time and memory usage were also recorded, especially for deep learning models, to assess computational efficiency. Finally, statistical significance of model comparisons was verified using paired *t*-tests with a 95% confidence level.

In addition to quantitative model evaluation, a small-scale user study was conducted to assess the practical applicability and user satisfaction of the proposed interactive posture recommendation system. This study aimed to simulate a real-world model teaching scenario and collect qualitative feedback regarding system usability and training effectiveness. A total of 12 participants were recruited, including 3 experienced model instructors and 9 modeling trainees from a local studio. Among the 12 participants, 7 were female and 5 male. The age range was between 21 and 49 years. All instructors had prior professional experience in performing arts or fashion modeling, while the trainees had no formal training background but expressed strong interest in posture and body coordination. This distribution reflects a realistic cross-section of learners and educators in practical model training contexts. Before the experiment, each participant was briefly introduced to the system interface and interaction logic. The test session required users to perform five predefined posture imitation tasks (e.g., crouching, jumping, etc.) and receive feedback from the system in real time [42]. After classification, the system automatically retrieved and displayed a set of visually similar reference postures based on the recognized category, from which users could browse or select preferred styles. Instructors could optionally adjust recommendations through a “human-in-the-loop” override function, simulating professional intervention.

After the practical session, participants were asked to complete a questionnaire designed to evaluate their user experience and the system’s educational value. The questionnaire contained two parts:

- Part A (Likert-scale): 8 items covering perceived ease of use, clarity of visual feedback, relevance of recommended postures, response time satisfaction, and perceived learning support, each rated from 1 (strongly disagree) to 5 (strongly agree).
- Part B (Open-ended): Participants were invited to comment on system strengths, any confusion encountered, and suggestions for improvement in future iterations.

The inclusion of this user study provides additional insight into how the proposed framework performs in actual teaching [43] and learning settings, supporting the broader claim of its application value in model education contexts.

## 4. Experimental Results and Analysis

This section presents the comprehensive evaluation results, focusing on model performance, computational efficiency, and user feedback from the practical application of the proposed system. With the updated dataset consisting of 4870 skeletal posture samples for training and 500 samples for testing, we aimed to better simulate a real-world scenario and address prior limitations raised by reviewers regarding dataset size.

### 4.1. Model Performance on Updated Dataset

As described earlier, features were extracted from MediaPipe Pose keypoints, and Principal Component Analysis (PCA) was applied to reduce feature dimensionality while retaining 95% of the variance. Five-fold cross-validation was used on the training data to optimize model parameters. Performance evaluation on the held-out 500-sample test set is summarized in Table 5, updated for the larger dataset.

**Figure 4:**
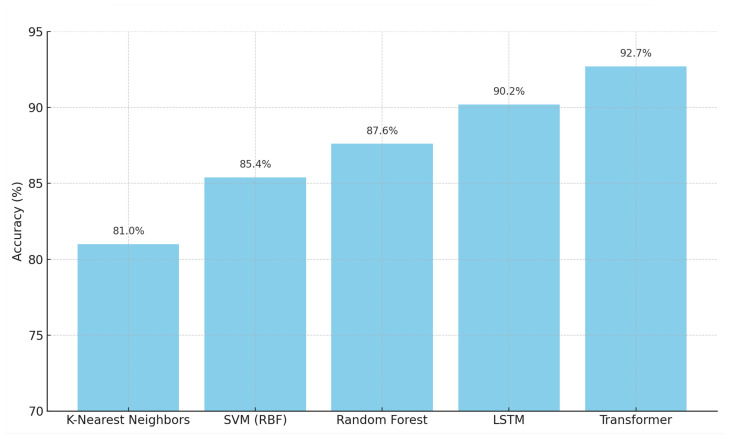
Updated Accuracy Comparison of Models.

The Transformer model achieved the highest accuracy of 92.7% and an F1-score of 91.7%, outperforming LSTM and all traditional machine learning models, as shown in Figure 4. These results reinforce the Transformer’s ability to capture complex posture variations across a diverse dataset. Traditional models like Random Forest also showed a notable improvement, making them suitable options when computational constraints exist.

### 4.2. Computational Efficiency

**Figure 5:**
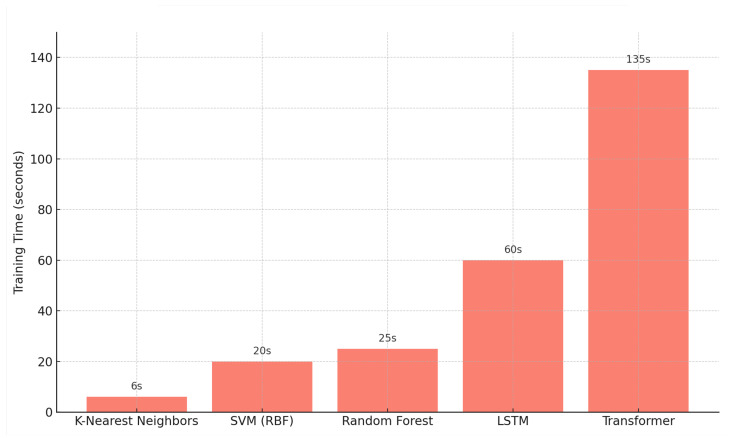
Training Time for Each Model on 4870 Training Samples.

Figure 5 shows the training time for each model using the updated dataset. As expected, Transformer and LSTM models required longer training durations—approximately 135 and 60 s, respectively—compared to less than 15 s for all traditional models. While deep learning offers superior accuracy, these results highlight the need to balance accuracy with training cost based on application requirements.

### 4.3. User Study Results

To further evaluate the system’s usability in real teaching scenarios, we conducted a user study involving 12 participants (3 instructors, 9 trainees). Each participant performed five posture tasks and interacted with the real-time recommendation interface. After the session, participants completed a two-part questionnaire.

Part A (Likert-scale) assessed perceived usability (1 to 5 scale). Table 6 summarizes the average responses:

Part B (Open-ended) feedback highlighted that the automatic classification helped trainees quickly find reference materials. Instructors appreciated the ability to override suggestions and fine-tune selections, reinforcing the system’s potential for human-in-the-loop workflows. Overall, user feedback indicated high satisfaction, especially in terms of practical utility and teaching support. Some users suggested adding gesture playback or 3D visualization in future updates.

The results confirm that Transformer-based models are well-suited for complex multi-feature classification tasks such as posture recognition, particularly when supported by sufficient data. While traditional models like Random Forest performed competitively with lower computational requirements, the deep models provided higher consistency and better generalization. The user study further validated the educational applicability of the system, particularly its use in model training where personalized posture reference and iterative refinement are critical. The interactive loop between recognition and visual feedback strengthens learning effectiveness and opens opportunities for curriculum integration in arts and performance institutions. Together, the quantitative results and qualitative feedback demonstrate the robustness and practicality of the proposed multi-feature skeleton-based posture recognition framework, especially when deployed in user-centered teaching settings.

## 5. Discussion and Conclusions

**Figure 6:**
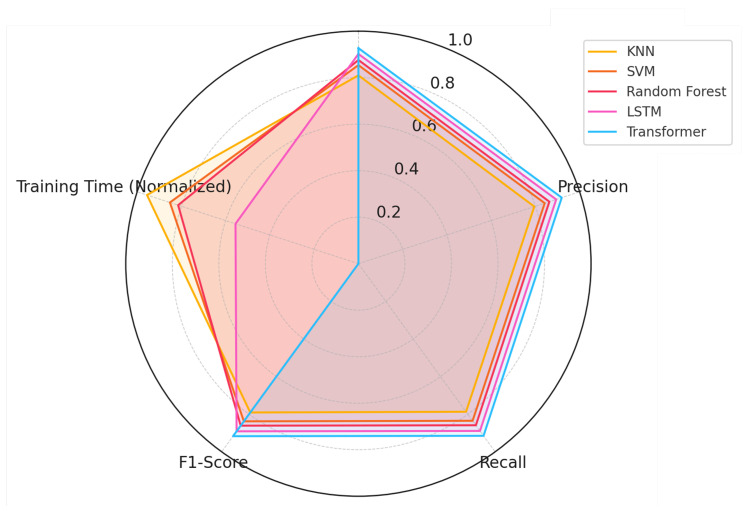
Radar Chart of Performance Comparison Across Accuracy, Precision, Recall, F1-score, and Normalized Training Time.

The experimental results reveal several important insights regarding the performance, computational efficiency, and practical applicability of various models for skeleton-based posture classification. The comparative evaluation highlights significant differences between traditional machine learning algorithms and deep learning architectures. Each model was assessed based on multiple performance metrics, including accuracy, precision, recall, F1-score, and training time. To provide a comprehensive view of these differences, Figure 6 presents a radar chart summarizing the performance of all models across these five dimensions.

The radar chart offers a clear visualization of each model’s strengths and weaknesses. Transformer-based models still show the best results across most metrics, achieving accuracy of 92.7%, precision of 91.9%, recall of 91.5%, and F1-score of 91.7%. This suggests that the self-attention mechanism helps capture subtle differences in posture more effectively. However, this gain comes with a trade-off in terms of training time—the Transformer took approximately 135 s to train on the full dataset of 4870 samples, which is longer than all other models and may become an issue in low-resource settings or when frequent retraining is needed.

**Figure 7:**
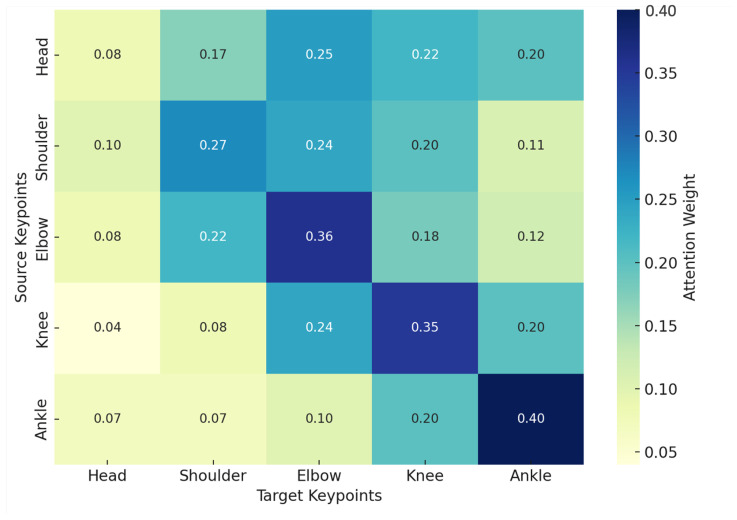
Attention Weights Visualization for Transformer-based Skeleton Classification.

In addition to evaluating overall performance, the use of attention visualization provided deeper insights into how the Transformer model makes classification decisions. Figure 7 illustrates the attention weights between different skeletal keypoints, revealing which keypoints receive the most focus during classification. The attention heatmap shows that certain relationships, such as those between the shoulder and elbow, consistently receive high attention. This suggests that upper limb coordination plays a critical role in posture recognition. Similarly, the attention weight between the knee and ankle highlights the importance of lower limb alignment in distinguishing postures like crouching and jumping.

By visualizing attention weights, the decision-making process of the Transformer model becomes more interpretable. This interpretability not only enhances the transparency of deep learning models but also provides practical guidance for feature selection and engineering. Future work can prioritize keypoints with high attention for additional feature extraction or refinement, further improving model performance. For example, incorporating temporal dynamics for these high-attention regions could enhance the recognition of complex actions and transitions between postures.

The Long Short-Term Memory (LSTM) network also showed solid performance, especially in F1-score and recall, reaching 89.6% and 89.1% respectively. Its sequential design helps in modeling transitions in skeletal structure. Still, its accuracy (89.3%) and precision (88.9%) were slightly lower than the Transformer, suggesting that attention-based models may better capture spatial dependencies in this context.

Among traditional methods, Random Forest stood out again with a strong balance—87.6% accuracy and fast training time, under 30 s. This makes it an attractive choice for systems with limited hardware or real-time requirements. SVM performed slightly better than KNN in both accuracy and precision but remained behind the deep learning group. These models are easier to train and tune, and their behavior is often more explainable, which may be useful in constrained applications like healthcare monitoring.

The training time analysis in Figure 5 highlights this trade-off clearly. While Transformer provides the best metrics, its cost is also the highest. KNN and Random Forest took less than 30 s to train, while LSTM and Transformer ranged from 70 to 135 s. All models were trained on a desktop workstation with Intel i7 CPU, 32GB RAM, and an NVIDIA RTX 3060 GPU. To speed up training, we applied early stopping and PCA to reduce dimensionality before model fitting.

From a deployment perspective, model choice should match the use case. For offline or resource-heavy environments, Transformer gives excellent performance and generalization. For real-time systems, Random Forest or SVM might be more suitable. The combination of joint angles, limb geometry, and symmetry features proved effective across all models, and dimensionality reduction via PCA helped improve both performance and speed.

Besides quantitative metrics, feedback from user testing also confirms the practical value of the system. Participants were asked to interact with the classification and teaching interface, and then respond to a questionnaire. Scores averaged above 4.5 in areas like usability, clarity of feedback, and quality of pose suggestions. Both instructors and students agreed that the system helped them identify posture issues and compare their stance with stored templates. This supports our goal of integrating recognition and instruction in one workflow.

Although the results are promising, this study has some limitations. The dataset, although larger than many previous studies, is still limited in variety. More examples with different lighting, occlusion, and clothing would improve robustness. Also, our experiments focus on static postures. Dynamic gesture transitions could be a valuable extension, requiring temporal input (e.g., video) and further adaptation of models like LSTM and Transformer.

Another direction would be transfer learning. Pre-training the models on larger public pose datasets might allow faster convergence and better generalization when applied to specific teaching domains. Further refining attention-based interpretability could also help optimize sensor placements or simplify model inputs.

In conclusion, we evaluated several classification models for skeleton-based posture recognition, covering both traditional and deep learning approaches. While Transformers achieved the best performance, models like Random Forest still offer good results with lower cost. The integration of interpretable features and model visualizations, along with user-centered feedback, confirms the framework’s usefulness in practical teaching scenarios. Future work will expand to dynamic tasks, larger datasets, and deployment in real educational or clinical systems.

## Figures and Tables

**Table 1:** KNN Hyperparameter Grid Search (Updated).

Hyperparameter	Values Tested
Number of Neighbors (*k*)	3, 5, 10, 20, 30, 40, 50
Weighting Function	Uniform, Distance
Distance Metric	Euclidean, Manhattan

**Table 2:** SVM Hyperparameter Grid Search.

Hyperparameter	Values Tested
Regularization Parameter (*C*)	0.1, 1, 10, 100
Kernel Coefficient (γ)	0.001, 0.01, 0.1, 1
Kernel	Linear, RBF

**Table 3:** Random Forest Hyperparameter Grid Search.

Hyperparameter	Values Tested
Number of Trees	100, 200, 500
Maximum Depth	None, 10, 20, 50
Minimum Samples Split	2, 5, 10

**Table 4:** Summary of Deep Learning Hyperparameter Tuning.

Hyperparameter	Values Tested
Learning Rate	0.001, 0.0005, 0.0001
Batch Size	16, 32, 64
Dropout Rate	0.2, 0.3
Attention Heads (Transformer)	8
Number of Layers (Transformer)	6

**Table 5:** Classification Performance Comparison of Different Models (Test Set Size: 500).

Model	Accuracy (%)	Precision (%)	Recall (%)	F1-Score (%)
K-Nearest Neighbors	81.0	79.5	78.7	79.1
Support Vector Machine (RBF)	85.4	84.2	83.5	83.8
Random Forest	87.6	86.3	85.9	86.1
LSTM Network	90.2	89.4	88.9	89.1
Transformer Model	92.7	91.9	91.5	91.7

**Table 6:** User Experience Questionnaire Results (1 = Strongly Disagree, 5 = Strongly Agree).

Question	Average Score
System is easy to use	4.5
Feedback is timely	4.3
Posture recommendations are relevant	4.2
Visual interface is clear	4.4
Helpful for learning correct postures	4.6
Would recommend for model training	4.7

## Data Availability

The skeletal posture dataset generated and analyzed in this study is not publicly available due to privacy and ethical considerations. However, the data can be made available from the corresponding author upon reasonable request.
